# A nationwide survey of antimicrobial stewardship infrastructure and CDC core elements in the Dominican Republic

**DOI:** 10.1017/ash.2025.10120

**Published:** 2025-08-29

**Authors:** Elianet Castillo, Rita Rojas-Fermin, Claudia Blanco, Antonio Villegas, Yeison Reyes, Arzina Aziz-Ali, Alfredo J. Mena Lora

**Affiliations:** 1 Infectious Diseases Department, CEDIMAT, Santo Domingo, Dominican Republic; 2 Hospital General Plaza de la Salud, Santo Domingo, Dominican Republic; 3 University of Illinois Chicago, Chicago, IL, USA

## Abstract

Antimicrobial resistance is a global threat, and antimicrobial stewardship programs (ASP) are vital to curb resistance. A survey of 20 Dominican Republic hospitals revealed ASPs were absent in 50% and compliance with CDC core elements varied, highlighting significant challenges and areas of opportunity in implementing effective stewardship in resource-limited settings.

## Introduction

Antimicrobial resistance (AMR) is one of the most pressing public health challenges, threatening our ability to effectively treat a wide range of infections caused by multidrug-resistant pathogens.^1^ In 2019, over 2 of every 5 infection-related deaths in the Americas were associated with AMR, underscoring its significant health and economic burden.^2^ According to the Pan American Health Organization, 34 countries in Latin America and the Caribbean are actively developing national action plans to combat AMR, which include the establishment and implementation of antimicrobial stewardship programs (ASPs).^3^ ASPs are recognized as essential tools for optimizing antimicrobial use (AU) and curbing resistance.^3^ However, in many low- and middle-income countries, the development and implementation of ASPs face challenges such as resource constraints, limited regulations, and over-the-counter access to antimicrobials.^4,5^ These barriers have been exacerbated by the increased AU during the COVID-19 pandemic, further contributing to the rise of AMR in these settings. Despite growing global attention on AMR and ASP, data on the implementation and effectiveness of ASPs in Latin America and the Caribbean remains limited. This study aims to address this gap by describing the presence and practices of ASPs in the Dominican Republic (DR).

## Methods

### Study design and participants

We conducted a cross-sectional survey of infectious disease (ID) specialists working in acute care hospitals in the DR to assess ASP in acute care settings. An anonymous web-based survey was developed in Google Forms to capture data. The survey, adapted from the Centers for Disease Control and Prevention (CDC) Infection Control Assessment and Response (ICAR) tool for antimicrobial stewardship, was distributed via professional society mailing lists between March 4 and April 24, 2024. Respondents were asked to evaluate ASPs based on their activities during the 12 months preceding the survey completion date.

### Data analysis

Data were tabulated, and descriptive statistical analyses were performed. The ICAR tool comprises a series of questions designed to evaluate antibiotic stewardship policies and activities, focusing on the implementation of the CDC’s core elements of ASPs: Leadership Commitment, Accountability, Drug Expertise, Action, Tracking, Reporting, and Education.

### Ethics

This study was approved by the Ethical Investigation Committee of CEDIMAT in March 2024.

## Results

### Hospital characteristics

**Figure 1.**
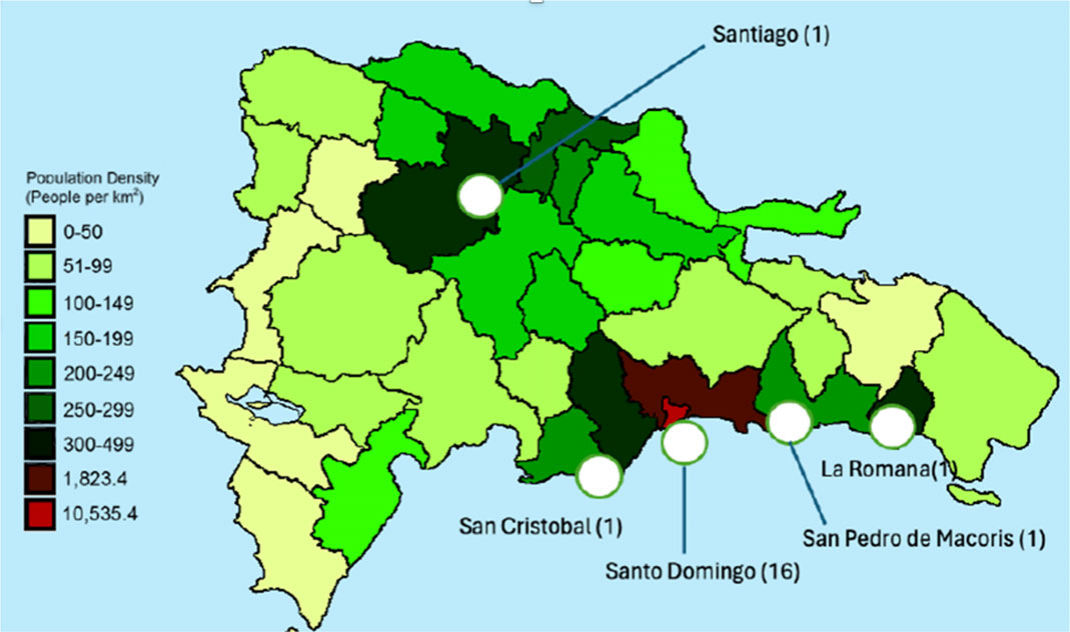
Population density of the Dominican Republic and geographic distribution of hospitals surveyed.

A total of 20 hospitals completed the survey, with 16 (80%) located in Santo Domingo and 1 each in La Romana, San Cristobal, San Pedro, and Santiago (1 (5%) each; Figure 1). Among these, 18 (90%) were tertiary care centers. ASPs were absent in 10 (50%) hospitals, present in 7 (35%), and in the process of development in 3 (15%). In 7 (35%) of 20 facilities, ASP was multidisciplinary and included pharmacy, ID, and hospital epidemiology. Among the 20 hospitals surveyed, 12 (60%) had paper and 8 (40%) electronic medical records. “Documentation of Indication” and “Center-Specific Treatment Guidelines” were reported by 5 (71%) of hospitals.

### Antimicrobial stewardship programs and compliance with CDC core elements

**Figure 2.**
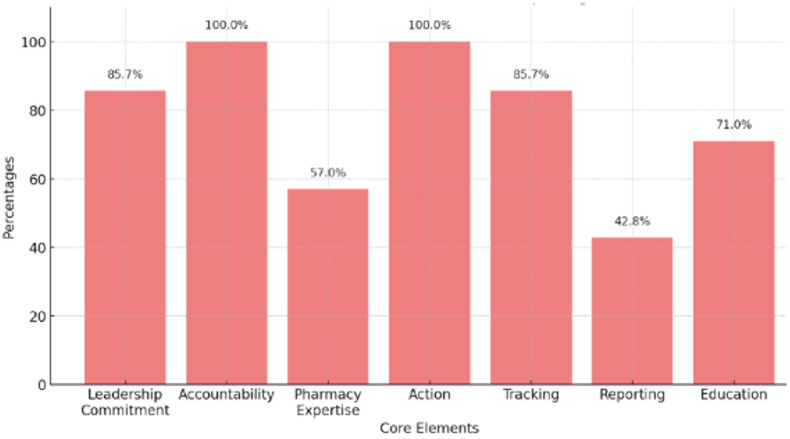
CDC Antimicrobial Stewardship Core Elements met by hospitals in the Dominican Republic.

Compliance with the CDC’s core elements for ASPs varied across hospitals. Among those with ASPs (N = 7), accountability and action were present in all (7 (100%)), leadership commitment and tracking were present in 6 (85.7%), and education in 5 (71.0%) (Figure 2). Pharmacy expertise and reporting were less common, at 4 (57.0%) and 3 (42.8%), respectively. Preauthorization and prospective audit and feedback (PAF) were also common, each implemented in 4 (57%) of hospitals. Less frequently reported interventions included “Antibiotic Timeout” and “Assessment of Penicillin Allergy,” both present in 1 (14%) hospital, and “Review of Outpatient Parenteral Antibiotic Therapy,” reported by 2 (29%).

## Discussion

ASPs were absent in half of the hospitals surveyed, and those with an ASP in place often lacked full implementation of the CDC core elements. Among DR hospitals with ASPs, 85.7% demonstrated leadership commitment and 100% accountability, differing from studies in the United States and other Latin American regions. Financial support for ASP was not specified. Over 95% of hospitals have established ASPs in the United States.^7^ In Latin America, a 2015 survey of 27 hospitals across 10 countries found that 59.0% had a formal written statement supporting ASPs.^8^ A 2022 study by Fabre *et al.*, surveying 20 hospitals across 5 Latin American countries, found that 67% of ASPs had leadership support through a written document and a designated individual responsible for the program, while a 2023 review of ASPs in 7 Latin American countries identified hospital leadership support (57%) and monitoring/communication (54.4%) as the least developed core elements.^6,7^

Common interventions in DR hospitals with ASPs included documentation of indication and hospital-specific treatment guidelines, both implemented by 71% of hospitals. Less frequently adopted interventions included antimicrobial restriction and preauthorization (57%), PAF (57%), review of outpatient parenteral antibiotic therapy (29%), antibiotic time-out (14%), and assessment of beta-lactam allergies (14%). Comparatively, the 2015 Munoz *et al.* survey reported that only 37% of hospitals performed antibiotic time-out audits after 48 hours, although 74.1% required preauthorization of specific antimicrobials.^8^ Tracking of antibiotic prescribing and monitoring was present in 85.7% of hospitals, and 71.0% fulfilled the Education core component by providing training on optimal antibiotic prescribing, side effects, and AMR within the last year. In contrast, the Fabre *et al.* study reported that only 35% of hospitals provided healthcare workers with education on ASP principles upon hiring.^9,10^ Pharmacy expertise, such as the presence of a co-leader for the ASP, was reported in 57% of hospitals, while reporting of antibiotic usage data to prescribers was performed in only 42% of hospitals. Major barriers for effective ASP remain. Pharmacists are unable to formally specialize in the DR. Widespread over-the-counter access to antimicrobials in the DR, including at pharmacies and bodegas, contributes significantly to antimicrobial misuse and may limit the impact of hospital-based ASP.^4^

### Limitations

This study has some limitations. It utilized a convenience sample of hospitals associated with ID physicians’ practices, which may not fully represent all hospitals nationwide. Additionally, the use of a modified survey instrument may have excluded other methods of ASP implementation. Despite these limitations, the survey provides valuable insights into the current state of ASPs in the DR and characterizes the actions implemented in hospitals with established programs.

## Conclusions

This study highlights the urgent need to scale up ASPs across the DR while addressing implementation deficits that require further support and engagement. Future studies should evaluate the impact of ASPs on reducing inappropriate AU, curbing the emergence of AMR, and lowering costs in hospitals.
